# Cod Residual Protein Prevented Blood Pressure Increase in Zucker fa/fa Rats, Possibly by Inhibiting Activities of Angiotensin-Converting Enzyme and Renin

**DOI:** 10.3390/nu10121820

**Published:** 2018-11-22

**Authors:** Iselin Vildmyren, Aslaug Drotningsvik, Åge Oterhals, Ola Ween, Alfred Halstensen, Oddrun Anita Gudbrandsen

**Affiliations:** 1 Dietary Protein Research Group, Department of Clinical Medicine, University of Bergen, 5021 Bergen, Norway; iselin.vildmyren@uib.no (I.V.); aslaug.drotningsvik@uib.no (A.D.); 2 K. Halstensen AS, P.O. Box 103, 5399 Bekkjarvik, Norway; alfred.halstensen@uib.no; 3 TripleNine Vedde AS, 6030 Langevåg, Norway; 4 Nofima AS, P.B. 1425 Oasen, 5844 Bergen, Norway; Aage.Oterhals@Nofima.no; 5 Møreforsking Ålesund AS, P.O. Box 5075, 6021 Ålesund, Norway; ola.ween@moreforsk.no; 6 Department of Clinical Science, University of Bergen, 5021 Bergen, Norway

**Keywords:** fish protein, fish meal, cod, rest raw material, hypertension

## Abstract

Hypertension is the leading risk factor for cardiovascular disease, and prevention of high blood pressure through diet and lifestyle should be a preferred approach. High intake of fish is associated with lower blood pressure, possibly mediated through the proteins since peptides with angiotensin-converting enzyme (ACE) inhibiting capacities have been identified in fish skin, backbone, and fillet. The effects of cod meals made from residual materials and fillet on blood pressure were investigated in obese Zucker fa/fa rats which spontaneously develop high blood pressure. Rats were fed diets containing water-soluble (stickwater) or water-insoluble (presscake) fractions of protein-rich meals from cod residual materials (head, gut, backbone with muscle residuals, skin, trimmings) or fillet. Rats were fed diets containing 25% of total protein from cod meal and 75% of protein from casein, or casein as the sole protein source (control group) for four weeks. Results show that a diet containing residual presscake meal with high gut content prevented blood pressure increase, and this cod residual meal also showed the strongest in vitro inhibitions of ACE and renin activities. In conclusion, a diet containing water-insoluble proteins (presscake meal) with high gut content prevented increase in blood pressure in obese Zucker fa/fa rats.

## 1. Introduction

The prevalence of hypertension is increasing worldwide, and is estimated to affect 1.56 billion adults by the year of 2025 [1]. Hypertension is the leading risk factor for cardiovascular disease [2], and the goal of prevention of hypertension is to avoid premature cardiovascular disease [3] since hypertension increases the risk of atherosclerosis, stroke, myocardial infarction, heart failure, peripheral vascular disease, disability, and damage to major organs such as heart and kidneys [4,5]. Hypertension can be prevented through dietary changes such as reduced sodium intake; maintaining an adequate intake of potassium; and consuming a diet low in saturated and total fat and rich in fruits, vegetables, whole-grain and low-fat dairy products [6]. Prevention of hypertension through the diet should be a preferred approach and more knowledge on effects of various nutrients on blood pressure is warranted.

Blood pressure is controlled by several mechanisms, of which the renin-angiotensin system may be the best known. Angiotensinogen is cleaved by renin to the biologically inactive angiotensin I, which is further converted to the vasoconstrictor angiotensin II by the angiotensin-converting enzyme (ACE). The cleavage of angiotensinogen to angiotensin I by renin is considered to be the rate determining step in the generation of angiotensin II [7]. ACE also catalyzes the degradation of the vasodilator bradykinin to inactive peptides, thus providing a second way of regulating blood pressure through manipulation of ACE activity [7], and a wide range of synthetic drugs targeting ACE-inhibition has been developed and are commonly used by hypertensive patients all over the world. Other factors that may influence blood pressure include arginine which is a precursor for the formation of the vasodilator nitric oxide [8], and dietary sodium which acts as a vasoconstrictor through its control of blood volume by increasing arterial constriction and peripheral vascular resistance [9] and effects on renin [10].

High intake of fish is reported to be associated with lower blood pressure in both clinical [11,12,13,14,15] and rat [16,17] studies, whereas some studies show no association between fish consumption and blood pressure [18,19,20]. A possible blood pressure lowering effect of fish may be mediated through the proteins in fish, as peptides with ACE-inhibiting capacities in vitro have been identified in fish fillet, skin, and backbone [21,22,23,24]. In addition, studies in spontaneously hypertensive rats have shown anti-hypertensive effects of ACE-inhibitory peptides identified in marine sources [25,26,27], however little is known of the potential effects of fish proteins on renin activity. Large amounts of protein-rich fish residual materials such as head, gut, bones, trimmings, and other cut-offs are produced by the world’s fisheries and aquaculture industries, but very little is used for human consumption [28]. Therefore, fish proteins from both fillet and residual materials as food component are of interest for regulation of blood pressure and thus for prevention of cardiovascular disease and should be investigated in vivo.

The obese Zucker fa/fa rat presents a range of abnormalities similar to those seen in humans with obesity—including insulin resistance, dyslipidemia, mild glucose intolerance, and hypertension—and is a popular rat model for studies of metabolic complications and possible treatments of obesity [29]. The Zucker rat is a valuable experimental model for hypertension as this rat develops an age-related increase in blood pressure, which is also the case for humans [30], and in Zucker fa/fa rats this development starts already before the age of 10 weeks [31,32]. The most used rat model for studies on development and treatment of hypertension is the spontaneously hypertensive rat, although this rat is representative for only a rare subtype of human hypertension; primary hypertension that is inherited in a Mendelian fashion [33]. The Zucker fa/fa rat could therefore be a more relevant model where hypertension co-exists with obesity and other metabolic disturbances in a real-life setting [31].

The aim of the present study was to investigate the effects of diets containing protein-rich meals from a selection of water-soluble and water-insoluble cod residuals or cod fillet on the development of high blood pressure in obese Zucker fa/fa rats which spontaneously develops high blood pressure, compared with Zucker fa/fa rats fed a control diet devoid of fish. Circulating nitrite + nitrate (as a measure of nitric oxide) and renin were measured to elucidate possible mechanisms involved in blood pressure regulation that were affected by the cod meals, and the in vitro potential of the dietary proteins to inhibit ACE and renin activities were investigated. Our hypothesis was that cod meal prepared from residual materials and fillet would prevent or delay the development of high blood pressure in obese Zucker fa/fa rats, possibly through inhibition of the renin-angiotensin system.

## 2. Materials and Methods

### 2.1. Ethical Approval

The animal experiment was approved in accordance with the Norwegian regulation on animal experimentation (approval no 11603). The protocol was approved by the Norwegian State Board of Biological Experiments with Living Animals.

### 2.2. Design

Thirty-eight male Zucker fa/fa rats (Crl:ZUC(Orl)-Lepr fa, from Charles River Laboratories, Calco, Italy) were randomly assigned to six experimental groups with six rats in each of the groups receiving cod meal, with eight rats in the control group. Groups had comparable mean body weight at baseline. The rats were housed in pairs in individually ventilated cages (IVC type 4, blue line from Tecniplast, Buguggiate, VA, Italy) with plastic housing, under standard conditions with a temperature of 21 ± 1 °C, and a light-dark cycle of 12 h. The intervention period started after at least one week of acclimatization under these conditions, i.e., when the rats were 9–11 weeks old and weighing 339 ± 14 g.

The intervention period was four weeks, and rats had ad libitum access to feed and tap water. Freshly thawed feed was provided daily. Systolic and diastolic blood pressures of conscious rats were measured at baseline (Day 0), on Day 14 and three days before endpoint (i.e., on Day 25). Rats were prewarmed in a heating cabinet at 32 °C for 30 min before blood pressure was measured 10 times using the tail-cuff method (CODA™ Non-Invasive Blood Pressure System, Kent Scientific Corporation, Litchfield, CT, USA). Mean arterial pressure (MAP) was calculated as (diastolic blood pressure + 1/3 (systolic blood pressure − diastolic blood pressure)).

Rats were housed individually for 24 h in metabolic cages without fasting in advance to evaluate feed and water intake, and urine volume. To allow for the rats to recover after housing in the metabolic cages before measurements of end-point blood pressure on Day 25, the rats were housed in metabolic cages on Day 17.

At the end of the feeding period (after four weeks of intervention), after a 12 h fast with access to tap water, the rats were euthanized while under anaesthesia with isoflurane (Isoba vet, Intervet, Schering-Plough Animal Health, Boxmeer, The Netherlands) mixed with oxygen. Blood was drawn directly from the heart and was collected in BD Vacutainer SST II Advance gel tubes (Becton, Dickinson, and Company, Franklin Lakes, NJ, USA) for isolation of serum. Serum samples were stored at minus 80 °C until analysis. Staff handling the rats and conducting the analyses were blinded, and rats were handled and euthanized in random order.

### 2.3. Preparation of Fish Meals

Two Norwegian factory trawlers, Havstrand (Havstrand AS) and Granit (Halstensen Granit AS), prepared presscake fish meals on board from residual materials from Atlantic cod (Gadus morhua) fished in the Barents Sea in July 2016. Havstrand headed and gutted the cod. The heads were ground before mixing with gut, heated in a continuous cooker and mechanical pressed. The press liquid were run over a three-phase decanter centrifuge to remove oil and suspended solids. The decanter solids were mixed with the presscake and dried to a presscake meal (PC-H). The decanter liquid (stickwater) was immediately frozen for further processing on land; i.e., heated to 90 °C, centrifuged to remove residual solids and oil, concentrated and freeze dried (SW-H). A blend of stickwater + presscake meal was prepared, containing 20% of the total dry matter from stickwater from Havstrand (SWPC-H). Granit produced skin free cod fillets. The residuals (head, gut, backbone, skin, and trimmings) were ground before heated in a continuous cooker and mechanical pressed. The presscake was dried to a press cake meal (PC-G). Skin free fillets from Granit were grinded, heated to 80 °C and freeze dried to produce a fillet meal (FM-G). All experimental fish meals were ground on a Retsch ZM-1 centrifugal mill (Retsch GmbH, Haan, Germany) with a ring sieve aperture of 1 mm before analysis and inclusion in diets.

The residual cod meal from Havstrand comprises only head and gut and contains approximately twice as much liver compared to the residual meal from Granit which contains head, gut, backbone, skin, and trimmings (calculated from the official conversion factors from the Norwegian Directorate of Fisheries, www.fiskeridir.no).

### 2.4. Diets

The rats were fed experimental diets based on the AIN-93G recommendation for growing rats [34] with addition of 1.6 g methionine/kg diet as recommended by Reeves [35] (Table 1). All diets contained 20 wt % proteins, and casein was the sole protein source in the Control diet. Cod meal from residual material or fillet were added to the other diets in amounts providing 25 wt % of total protein from cod, while casein constituted the remaining 75 wt % of protein. Five diets containing different cod meals were prepared, containing either stickwater from Havstrand (SW-H), presscake meal from Havstrand (PC-H), a blend of stickwater + presscake meal from Havstrand (SWPC-H), presscake meal from Granit (PC-G), or fillet meal from Granit (FM-G).

Casein was purchased from Sigma-Aldrich (Munich, Germany). All feed ingredients except cod meals and casein were purchased from Dyets Inc. (Bethlehem, PA, USA). Since the feeds were given as powder formulas, the rats always had access to wood chewing sticks. The rats were weighed every seventh day during the intervention period. Casein and cod meals were not hydrolyzed prior to use.

### 2.5. Analyses of Diets

Total energy in diets was measured using an IKA C6000 global standards calorimeter in isoperibol measurement mode (IKA^®^-Werke GmbH & Co, Staufen, Germany). Total amino acids, taurine, fatty acids, sodium, potassium and chloride in diets were analyzed by Nofima BioLab (Fyllingsdalen, Norway). Total amino acid composition and content of cysteine + cystine were measured according to the method of Cohen & Michaud [36], and tryptophan was determined by the method of Miller [37]. Taurine was quantified by HPLC using the Waters Pico-Tag method as described by Bidlingmeyer et al. [38]. Lipids were extracted according to the AOCS method Ce 1b-89, and fatty acids were quantified as described by Oterhals & Nygård [39]. Contents of sodium and potassium in the diets were determined by flame atomic absorption spectrometry in accordance with ISO6869:2000 [40] using Perkin Elmer Analyst 400 with an AS 90plus autosampler (PerkinElmer, Waltham, MA, USA). Chloride in diets was determined by volumetric method in accordance with AOAC Official Method 937.09, by boiling the sample in silver nitrate and nitric acid to precipitate silver chloride. The residual soluble silver ions were titrated with ammonium thiocyanate [41].

### 2.6. In Vitro Inhibition of Angiotensin Converting Enzyme (ACE) and Renin

Casein and cod protein meals were added Trizma buffer (50 mM, pH 8.0) and hydrolyzed using trypsin from bovine pancreas (T1426, from Sigma-Aldrich) at 45 °C for 4 h as recommended by Shalaby et al. [42]. ACE-inhibition was measured using the method by Shalaby et al. [42], as previously described [43]. Renin inhibition was measured using the Renin Assay Kit (MAK157, from Sigma-Aldrich) as described in the user manual. Protein in hydrolysates were quantified on the Cobas c111 system (Roche Diagnostics GmbH, Mannheim, Germany) using the TP2 kit from Roche.

### 2.7. Analyses in Serum and Urine

As a measure of nitric oxide we measured the stable metabolites of nitric oxide metabolism, i.e., nitrite and nitrate, in serum using the Nitrite/Nitrate Assay Kit (cat #23479, Sigma-Aldrich, Munich, Germany) based on the Griess assay. Serum was filtrated (Amicon Ultra-0.5 Centrifugal Filter Unit with Ultracel membrane 10K device, Merck KGaA, Darmstadt, Germany) to remove hemoglobin and proteins before analysis of nitrite and nitrate. Sodium in urine was analyzed on the Cobas c111 system (Roche Diagnostics GmbH, Mannheim, Germany) using the Ion-Selective Electrode module from Roche Diagnostics.

### 2.8. Statistical Analyses

Variables were evaluated for normality by the Shapiro-Wilk test, Q-Q plots and histograms. Most variables were within normal distribution, and variables that were not normally distributed were log-transformed before parametric statistical tests were performed. Variables were compared between groups using one-way analysis of variance (ANOVA) with Fisher’s least significant difference (LSD) post-hoc test when appropriate. Changes in MAP from baseline to Day 14, and from baseline to endpoint within each group were tested using the paired sampled T-test, and the within-group changes were compared using ANOVA with LSD post-hoc test. Level of statistical significance were set at *p* < 0.05. Statistical analyses were performed using IBM SPSS Statistics 25 (SPSS, Inc., IBM Corporation, Armonk, NY, USA). Results from rat samples are presented as mean with standard deviations as a measure of variability. Results from in vitro inhibition of ACE and renin are presented as mean with standard error of mean as a measurement of the uncertainty of the mean measurements. Means with different letters are significantly different. One rat in the SW-H group had a lean phenotype and was excluded from all analyses, therefore results are presented for *n* = 5 rats in SW-H group, for *n* = 6 rats in the other cod meal diet groups, and for *n* = 8 rats in the control group.

## 3. Results

### 3.1. Measurements of Blood Pressure

**Figure 1:**
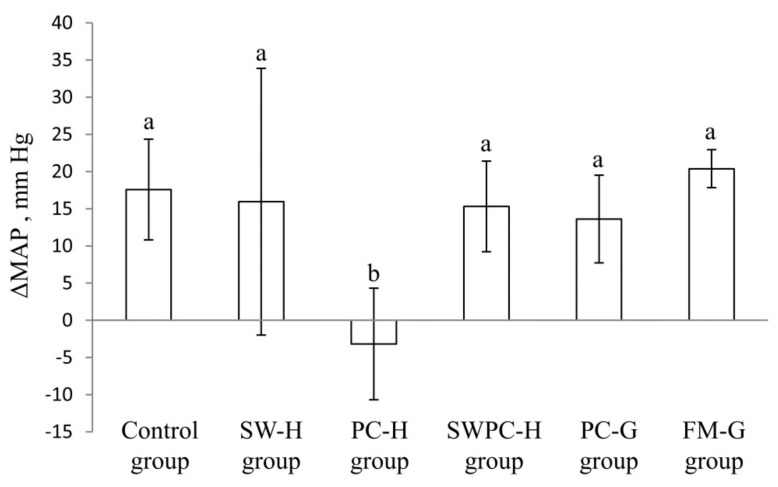
Changes in mean arterial pressure (MAP) from baseline to endpoint. Values are means, with standard error of mean represented as vertical bars. Values are shown for *n* = 8 in control group, *n* = 5 in SW-H, and *n* = 6 in all other groups. Groups are compared using one-way ANOVA with LSD post hoc test. Bars with different letters are significantly different (*p* < 0.05). BP; blood pressure, SW-H; stickwater from Havstrand, PC-H; presscake meal from Havstrand, SWPC-H; stickwater + presscake meal from Havstrand, PC-G; presscake meal from Granit, FM-G; fillet meal from Granit.

Systolic and diastolic blood pressures were measured in conscious rats at baseline, on Day 14 and three days before endpoint (i.e., Day 25). MAP was similar in all groups at baseline; mean (SD) for all rats was 114 (9) mm Hg with *p* ANOVA = 0.41. One-way ANOVA analyses show differences between the groups for MAP at Day 14 (*p* ANOVA 6.8 × 10^−4^) and at endpoint (*p* ANOVA 6.8 × 10^−4^). The changes in MAP from baseline to Day 14, and from baseline to endpoint were significantly lower in PC-H group when compared to all other groups (Figure 1 and Table 2). A paired samples T-test for PC-H group reveal that there were no statistically significant changes in MAP throughout the study period (*p* > 0.05), meaning that the blood pressures were not changed from baseline to endpoint in this group. Changes in MAP in SW-H, SWPC-H, PC-G and FM-G groups from baseline to Day 14, and from baseline to endpoint were not changed, and were similar to those of control group. At Day 14 the change in MAP from baseline was significantly different between PC-G and FM-G groups, but no difference in MAP was seen between these groups at endpoint.

### 3.2. Circulating Concentration of Nitrite + Nitrate

**Figure 2:**
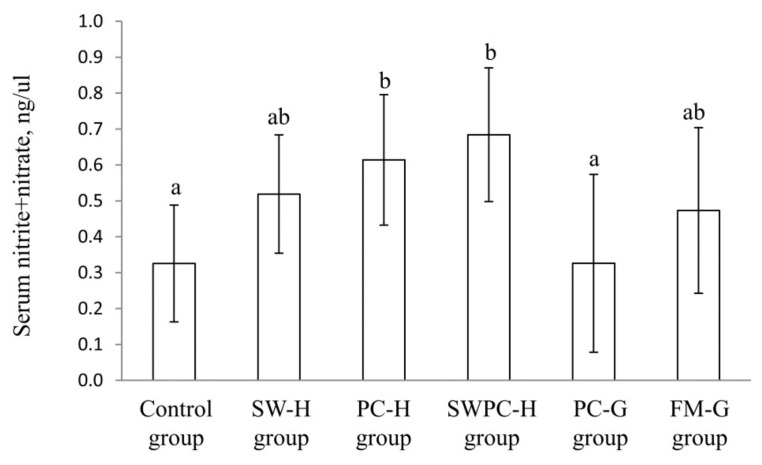
Serum concentration of nitrite + nitrate. Values are means, with standard error of mean represented as vertical bars. Values are shown for *n* = 8 in Control group, *n* = 5 in SW-H, and *n* = 6 in all other groups. Groups are compared using one-way ANOVA with LSD post hoc test when appropriate. Bars with different letters are significantly different (*p* < 0.05). SW-H; stickwater from Havstrand, PC-H; presscake meal from Havstrand, SWPC-H; stickwater + presscake meal from Havstrand, PC-G; presscake meal from Granit, FM-G; fillet meal from Granit.

Serum concentration of nitrite + nitrate (*p* ANOVA = 0.022) was significantly higher in PC-H and SWPC-H when compared to Control group (*p* values 0.015 and 0.0049, respectively), and in addition serum nitrite+nitrate concentrations were significantly higher in PC-H and SWPC-H when compared to PC-G (*p* values 0.022 and 0.0081, respectively), with no differences between the other experimental groups (Figure 2).

### 3.3. In Vitro Inhibition of ACE and Renin Activities

**Figure 3:**
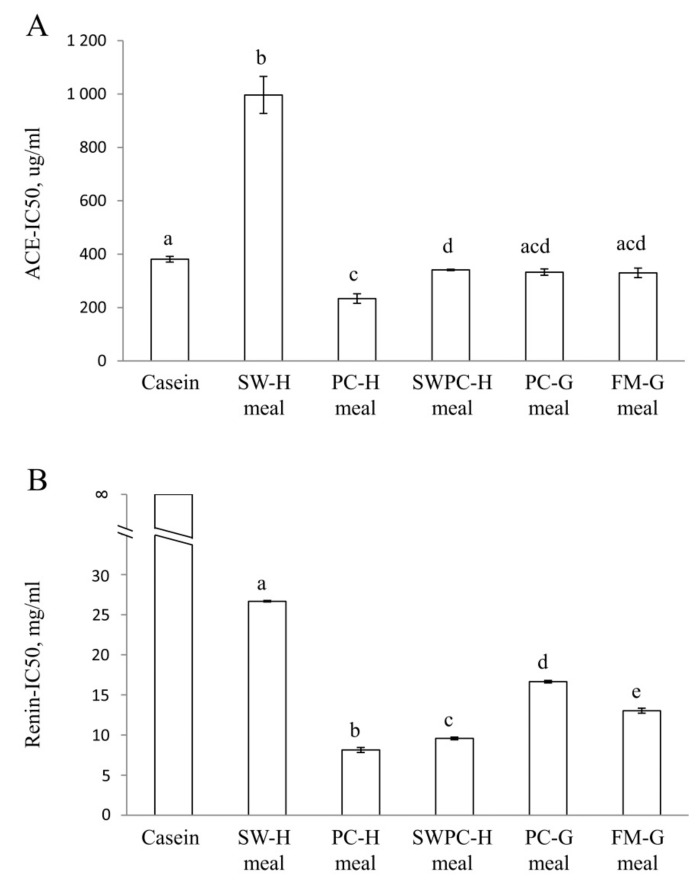
In vitro inhibition of activities of angiotensin-converting enzyme (**A**) and renin (**B**). Values are means, with standard error of mean represented as vertical bars. Values are shown for two or three measurements for casein and cod protein meals. Proteins are compared using one-way ANOVA with LSD post hoc test. Bars with different letters are significantly different (*p* < 0.05). SW-H; stickwater from Havstrand, PC-H; presscake meal from Havstrand, SWPC-H; stickwater + presscake meal from Havstrand, PC-G; presscake meal from Granit, FM-G; fillet meal from Granit.

The ACE and renin inhibiting capacities of the dietary proteins were measured after hydrolysis with trypsin by calculating IC50 concentrations. For ACE-inhibition (*p* ANOVA = 2.0 × 10^−5^), the ACE-IC50 for SW-H protein was much higher (i.e., less potent) when compared to all other groups (Figure 3A), whereas ACE-IC50 concentrations for both PC-H and SWPC-H proteins were lower when compared to casein. In addition, ACE-IC50 for PC-H protein was lower when compared to SWPC-H protein, with no differences in ACE-IC50 between the other dietary proteins. For renin activity inhibition, no measurable inhibition was detected for casein, therefore casein was not included in the ANOVA analysis (Figure 3B). All cod meal proteins had different renin-IC50 from each other (*p* ANOVA = 1.5 × 10^−7^), with the highest renin-IC50 observed for SW-H protein and the lowest renin-IC50 for PC-H protein.

### 3.4. Growth and Dietary Intake

All experimental groups had similar body weights at baseline, and no differences were seen in percent growth and body weight-to-square body length (without tail) ratio between the groups after four weeks’ intervention (Table 3). Some differences were seen between the groups for mean daily energy intake (*p* ANOVA = 0.035), with higher intake in FM-G group when compared to control group, PC-H group and PC-G group, and higher intake in SWPC-H group when compared to PC-H group and PC-G group. Water intake and urine output per 24 h were similar between all groups (*p* ANOVA 0.19 and 0.41, respectively, data not presented).

Daily intakes of amino acids, taurine, fatty acids and electrolytes are presented relative to bodyweight in the Table S1. Intakes of most compounds are different between the groups, as demonstrated by low *p*-values after one-way ANOVA testing and subsequent post-hoc LSD tests. Intakes of alanine, arginine, aspartic acid + asparagine and glycine were higher in groups fed diets containing cod meal when compared to the control diet, which contained casein as the sole protein source. Of special interest in the present study are intakes of arginine which is a precursor of the vasodilator nitric oxide [8], taurine which is shown to have hypotensive effect [44], the long-chain *n*-3 PUFAs which may delay development of hypertension [45,46] and sodium which may act as a vasoconstrictor [9]. From calculations of dietary intake it is evident that arginine intake was not particularly high and the sodium intake was not particularly low in the PC-H group, which experienced a delay in the blood pressure increase compared to the other groups. Taurine and long-chain *n*-3 PUFAs were not detected in the control diet, but the comparisons between groups fed diets with cod meal/proteins show that taurine intake was not especially high in PC-H group, however the *n*-3 PUFA intake was similar to SW-PC group and higher compared to SW-H, PC-G and FM-G groups.

### 3.5. Urine Sodium Excretion

**Figure 4:**
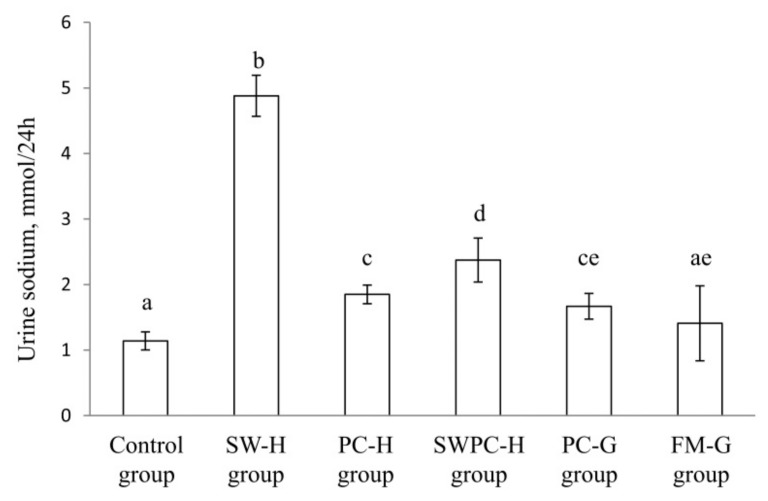
Urine 24 h sodium excretion. Values are means, with standard error of mean represented as vertical bars. Values are shown for *n* = 8 in Control group, *n* = 5 in SW-H, and *n* = 6 in all other groups. Groups are compared using one-way ANOVA with LSD post hoc test when appropriate. Bars with different letters are significantly different (*p* < 0.05). SW-H; stickwater from Havstrand, PC-H; presscake meal from Havstrand, SWPC-H; stickwater + presscake meal from Havstrand, PC-G; presscake meal from Granit, FM-G; fillet meal from Granit.

Urinary sodium excretion showed a large variation between the dietary groups (*p* ANOVA = 1.9 × 10^−18^). The mean sodium excretion in urine (per 24 h) was higher in SW-H group compared to all other groups (Figure 4). Urinary sodium excretion was similar in PC-H and PC-G groups, whereas rats fed SWPC-H had a urine sodium excretion that was between SW-H and PC-H, as could be expected since SWPC-H meal is a mixture of these two meals. Sodium excretion was similar in rats fed FM-G diet and those fed the control diet.

## 4. Discussion

In the present study we show for the first time that intake of cod presscake meal produced from residual materials with high content of gut prevented blood pressure increase in obese Zucker fa/fa rats. This effect was evident already after two weeks, and persisted until endpoint at four weeks. The other cod meal diets tested, containing either a residual stickwater, a mixture of residual presscake and stickwater, a residual presscake meal with low gut content, or fillet did not affect blood pressure development in this rat model. We chose to use obese Zucker fa/fa rats in this experiment, since this model resembles human obesity [29] and the rats spontaneously develop high blood pressure from a young age, that is, before the age of 10 weeks [31,32,47].

Renin and ACE are important enzymes for blood pressure control since they catalyze the conversion of angiotensinogen via angiotensin I to the vasoconstrictor angiotensin II, with renin as the rate determining enzyme [7]. Medications that inhibit either renin or ACE activity have been developed, although Skeggs et al. already in 1957 suggested that renin inhibition should be preferred to ACE inhibition [48]. Peptides with ACE inhibiting properties in vitro have been identified in fillet, skin, and backbone from various fish species [21,22,23,24] and in milk proteins such as casein [49]. Renin inhibitory effects of cod fillet has been demonstrated [50], however little is known about whether proteins from fish residual materials and milk may affect renin activity in vitro, and whether in vitro findings are transferable to live animals including humans.

In the present study we found that PC-H had significantly (although marginally) lower in vitro ACE-IC50 and renin-IC50 (i.e., the strongest ACE and renin inhibiting potencies) compared to casein and all other cod meals after trypsin digestion, and in line with this we found that the PC-H diet prevented the development of high blood pressure in the obese Zucker rats. This suggests that the postponed development of high blood pressure in PC-H fed rats could be regulated through the renin-angiotensin system; however, since ACE and renin activities are measured in vitro these may not be directly transferable to effects in the rats. We saw no associations between the in vitro ACE and renin inhibitory capacities and blood pressure development in the other experimental groups. The ACE-IC50 for FM-G meal, containing cod fillet meal, was not markedly different from the ACE-IC50 for casein, which was surprising since we recently demonstrated that proteins from cod fillet had a lower ACE-IC50 compared to a casein-whey (9:1) mixture [43].

ACE-IC50 and renin-IC50 were highest for SW-H, however the blood pressure development in rats fed SW-H diet was similar to the other experimental groups except for the PC-H group (where MAP was unchanged). Also, dietary sodium can act as a vasoconstrictor through its control of blood volume by increasing arterial constriction and peripheral vascular resistance [9]. Sodium intake was lowest in the control group, but still no difference was seen for blood pressure development between this group and SW-H, SWPC-H, PC-G, and FM-G groups. In the present study, the sodium intake was significantly higher in the SW-H diet compared to the other diets, and the high urinary output of sodium in rats fed this diet imply that the kidney coped well with this higher sodium load, and thus avoided development of hypervolemic high blood pressure.

Components other than sodium in the diets may also affect blood pressure development, such as arginine, taurine, and long-chain *n*-3 PUFAs. Arginine is a conditionally essential amino acid in rats [51], and is a precursor for the formation of the vasodilator nitric oxide [8]. It has been suggested by others that dietary fish proteins delays development of hypertension due to higher arginine content in fish proteins compared to casein [16]. We found no association between arginine intake, serum nitrite + nitrate concentration (as a measure of nitric oxide), and blood pressure development in the present study; however, we cannot exclude the possibility that the higher serum nitrite + nitrate in PC-H group compared to the control group may have beneficially contributed to the prevention of blood pressure increase in this group. Intake of taurine has been shown to have a hypotensive effect in prehypertensive humans [44] and to delay blood pressure increase in spontaneously hypertensive rats [52], but the mechanisms behind this is not fully elucidated. Since the taurine intake in the PC-H group was relatively low when compared to especially the SW-H diet, and only the former diet prevented an increase in blood pressure, the observed effects on blood pressure by the PC-H diet can probably not be ascribed to the taurine intake.

The gut residual includes liver in addition to intestines and other internal organs. Since the residual cod meal from Havstrand contains approximately twice as much liver compared to the residual meal from Granit, and since cod liver contains high amounts of long-chain *n*-3 PUFAs, the groups fed PC-H and SWPC-H diets had the highest intake of EPA, DPA, and DHA of the experimental diets. Studies in spontaneously hypertensive rats report a delay in the development of hypertension after intake of fish oil [45,46], and a negative association between fish fillet or fish oil intake and blood pressure has been observed in several studies [11,12,13,14,53,54]. A statement from the American Heart Association concludes that high doses of fish oil may reduce blood pressure in hypertensive individuals, but have no mention of fish oil or fish fillet intake for the prevention of development of high blood pressure [55]. In a recent paper [17] we show that feeding obese Zucker fa/fa rats a diet containing salmon fillet with low *n*-3 PUFA content prevented blood pressure increase, comparable to that observed in the PC-H group in the present experiment. Since only the PC-H diet prevented the blood pressure increase we cannot conclude if the dietary content of long-chain *n*-3 PUFA in PC-H diet played a role to prevent blood pressure increase in the present study.

There are some methodological limitations in the present study. This study was designed to investigate the effects of different cod meals on the development of high blood pressure using an experimental design that is relevant for human nutrition. We used a relevant rat model that develops high blood pressure and diets with 25% of protein from fish meal, and future studies should investigate if these findings are relevant for human hypertension. Blood pressure was measured using the non-invasive tail-cuff method (volume-pressure recording) at baseline and near endpoint of the intervention period instead of continuous intravascular blood pressure measured by telemetry, since comparison of these methods shows similar results in mice [56].

## 5. Conclusions

The present study demonstrates that presscake meal from cod residual materials with high gut content (PC-H) effectively prevented the increases in MAP that are normally observed in obese Zucker fa/fa rats, whereas the other cod meals tested did not influence the blood pressure development in these rats. In line with this, PC-H had the strongest effect on in vitro inhibition of ACE and renin activities of the cod meals tested.

## Figures and Tables

**Table 1:** Composition of the experimental diets.

g/kg Diet	Control Diet	SW-H Diet	PC-H Diet	SWPC-H Diet	PC-G Diet	FM-G Diet
Casein *	223.8	167.8	167.8	167.8	167.8	167.8
Stickwater from Havstrand ^†^	-	82.2	-	-	-	-
Presscake meal from Havstrand ^‡^	-	-	81.9	-	-	-
Stickwater + Presscake mealfrom Havstrand ^§^	-	-	-	84.6	-	-
Presscake meal from Granit ^||^	-	-	-	-	77.6	-
Fillet meal from Granit ^#^	-	-	-	-	-	60.7
Soybean Oil	70.0	70.0	70.0	70.0	70.0	70.0
Cornstarch	504.1	477.8	478.2	475.5	482.5	499.3
Sucrose	90.0	90.0	90.0	90.0	90.0	90.0
Cellulose	50.0	50.0	50.0	50.0	50.0	50.0
Tert-butylhydroquinone	0.014	0.014	0.014	0.014	0.014	0.014
Mineral Mix (AIN-93MX)	35.0	35.0	35.0	35.0	35.0	35.0
Vitamin Mix (AIN-93VX)	10.0	10.0	10.0	10.0	10.0	10.0
L-Methionine	1.6	1.6	1.6	1.6	1.6	1.6
L-Cystine	3.0	3.0	3.0	3.0	3.0	3.0
Choline Bitartrate **	2.5	2.5	2.5	2.5	2.5	2.5
Growth and Maintenance Supplement ^††^	10.0	10.0	10.0	10.0	10.0	10.0

* contains 89.4% crude protein; ^†^ contains 60.8% crude protein; ^‡^ contains 61.1% crude protein; ^§^ contains 59.1% crude protein; ^||^ contains 64.5% crude protein; ^#^ contains 82.3% crude protein; ** contains 41.1% choline; ^††^ contains vitamin B12 (40 mg/kg) and vitamin K1 (25 mg/kg) mixed with sucrose (995 g/kg) and dextrose (5 g/kg); SW-H; Stickwater from Havstrand, PC-H; Presscake meal from Havstrand, SWPC-H; Stickwater + Presscake meal from Havstrand, PC-G; Presscake meal from Granit, FM-G; Fillet meal from Granit.

**Table 2:** Mean arterial pressure (MAP) measured at baseline, Day 14 and at endpoint.

	Control Group	SW-H Group	PC-H Group	SWPC Group	PC-G Group	FM-G Group
MAP baseline, mm Hg	111 ± 11	114 ± 5	120 ± 8	115 ± 10	110 ± 7	112 ± 8
MAP Day 14, mm Hg	126 ± 10	127 ± 7	115 ± 13	128 ± 8	127 ± 12	118 ± 8
MAP endpoint, mm Hg	128 ± 10	130 ± 18	117 ± 7	130 ± 13	124 ± 9	132 ± 10
*p* for change baseline to Day 14	4.5 × 10^−4^	4.1 × 10^−3^	0.22	0.051	0.011	6.4 × 10^−3^
*p* for change baseline to endpoint	1.6 × 10^−4^	0.12	0.35	0.0016	2.4 × 10^−3^	6.5 × 10^−6^
ΔMAP baseline to Day 14, mm Hg	15 ± 7 ^ab^	12 ± 5 ^ab^	−5 ± 9 ^c^	13 ± 12 ^ab^	17 ± 11 ^a^	6 ± 3 ^b^
ΔMAP baseline to endpoint, mm Hg	18 ± 7 ^a^	16 ± 18 ^a^	−3 ± 8 ^b^	15 ± 6 ^a^	14 ± 6 ^a^	20 ± 3 ^a^

Data are presented as mean ± standard deviation, *n* = 8 in Control group, *n* = 5 in SW-H, and *n* = 6 in all other groups. Changes in MAP from baseline to Day 14, and from baseline to endpoint within each group were tested using the paired sampled T-test, and the within-group changes were compared using ANOVA with LSD post-hoc test. Means in a row with different letters are significantly different (*p* < 0.05). SW-H; stickwater from Havstrand, PC-H; presscake meal from Havstrand, SWPC-H; stickwater + presscake meal from Havstrand, PC-G; presscake meal from Granit, FM-G; fillet meal from Granit.

**Table 3:** Bodyweight at baseline and growth at time of euthanasia, and energy intake at day 17.

	Control Group	SW-H Group	PC-H Group	SWPC-H Group	PC-G Group	FM-G Group	*p* Anova
Bodyweight at baseline, g	339 ± 16	345 ± 15	340 ± 11	334 ± 14	335 ± 13	342 ± 16	0.81
Body weight gain, %	31 ± 6	37 ± 5	35 ± 3	37 ± 5	30 ± 4	34 ± 6	0.053
Bodyweight-to-square body length without tail ratio, kg/m^2^	8.4 ± 0.5	8.3 ± 0.4	8.3 ± 0.4	8.2 ± 0.4	8.3 ± 0.3	8.4 ± 0.3	0.94
Energy intake, kcal/kg bodyweight/24 h	219 ± 31 ^ab^	238 ± 10 ^abc^	209 ± 14 ^a^	245 ± 20 ^bd^	212 ± 36 ^a^	248 ± 25 ^cd^	0.035

Data are presented as mean ± standard deviation, *n* = 8 in Control group, *n* = 5 in SW-H, and *n* = 6 in all other groups; Groups are compared using one-way ANOVA with LSD post hoc test when appropriate; Means in a row with different letters are significantly different (*p* < 0.05); SW-H; stickwater from Havstrand, PC-H; presscake meal from Havstrand, SWPC-H; stickwater + presscake meal from Havstrand, PC-G; presscake meal from Granit, FM-G; fillet meal from Granit.

## Supplementary Materials

The following are available online at http://www.mdpi.com/2072-6643/10/12/1820/s1, Table S1: Daily intake of amino acids, taurine, fatty acids and electrolytes.
