# Supplementary material for: Cod Residual Protein Prevented Blood Pressure Increase in Zucker fa/fa Rats, Possibly by Inhibiting Activities of Angiotensin-Converting Enzyme and Renin
Source: Nutrients. 2018 Nov 22;10(12):1820. doi: 10.3390/nu10121820 (PMC6315726; doi:10.3390/nu10121820)
Supplement: Supplementary file 1 [file nutrients-10-01820-s001.pdf]

Supplemental Table S1: Daily intake of amino acids, taurine, fatty acids and electrolytes

mg/kg bodyweight/24h	Control group	SW-H group	PC-H group	SWPC-H group	PC-G group	FM-G group	*p* ANOVA
Alanine	297 ± 42[a]	468 ± 20[b]	391 ± 26[c]	446 ± 37[b]	375 ± 64[c]	455 ± 45[b]	8.9x10^{-8}
Arginine	332 ± 47[a]	435 ± 18[bc]	391 ± 26[b]	475 ± 39[c]	385 ± 66[b]	471 ± 47[c]	4.8x10^{-6}
Aspartic acid+asparagine	624 ± 89[a]	769 ± 33[b]	758 ± 51[b]	841 ± 70[b]	751 ± 128[b]	948 ± 95[c]	4.2x10^{-6}
Cysteine+cystine	198 ± 28[a]	223 ± 9[ab]	228 ± 15[bd]	263 ± 22[c]	224 ± 38[ade]	247 ± 25[bce]	0.0013
Glutamic acid+glutamine	2106 ± 301[ab]	2151 ± 91[ab]	1941 ± 130[a]	2272 ± 188[bc]	1955 ± 334[a]	2464 ± 246[c]	0.0056
Glycine	178 ± 25[a]	641 ± 27[b]	371 ± 25[c]	336 ± 37[d]	331 ± 57[cf]	309 ± 31[ef]	3.5x10^{-19}
Histidine	258 ± 37[ab]	245 ± 10[a]	243 ± 16[a]	286 ± 24[bc]	244 ± 42[a]	303 ± 30[c]	0.0039
Isoleucine	466 ± 67[ab]	440 ± 19[a]	441 ± 30[a]	515 ± 43[bc]	448 ± 77[a]	567 ± 57[c]	0.0013
Leucine	902 ± 129[ab]	864 ± 37[ab]	847 ± 57[a]	990 ± 82[bc]	843 ± 144[a]	1055 ± 106[c]	0.0049
Lysine	768 ± 110[a]	780 ± 33[ac]	748 ± 50[a]	887 ± 73[bc]	780 ± 133[ab]	1010 ± 101[c]	1.4x10^{-4}
Methionine	312 ± 45[a]	340 ± 14[a]	317 ± 21[a]	389 ± 32[b]	322 ± 55[a]	426 ± 43[b]	2.2x10^{-5}
Phenylalanine	500 ± 72[ab]	474 ± 20[ab]	470 ± 32[a]	538 ± 45[bc]	458 ± 78[a]	567 ± 57[c]	0.014
Proline	1060 ± 152[a]	1081 ± 46[a]	911 ± 61[b]	1105 ± 91[a]	897 ± 153[b]	1066 ± 107[a]	0.0081
Serine	590 ± 84[ab]	646 ± 27[ac]	565 ± 38[ab]	670 ± 55[c]	556 ± 95[b]	673 ± 67[c]	0.0099
Taurine*	<LOD	145 ± 6[a]	15 ± 1[b]	49 ± 4[c]	12 ± 2[b]	30 ± 3[d]	2.0x10^{-27}
Threonine	421 ± 60[ab]	418 ± 18[ab]	401 ± 27[a]	464 ± 38[bc]	395 ± 68[a]	483 ± 48[c]	0.019
Tryptophan	119 ± 17	123 ± 5	124 ± 8	126 ± 10	127 ± 22	140 ± 14	0.17
Tyrosine	421 ± 60[a]	407 ± 17[a]	376 ± 25[a]	424 ± 35[a]	370 ± 63[a]	488 ± 49[b]	0.0019
Valine	585 ± 84[ab]	574 ± 24[ab]	565 ± 38[a]	658 ± 54[bc]	570 ± 98[a]	707 ± 71[c]	0.0037
14:0	6.4 ± 0.9[a]	7.8 ± 0.3[b]	15.4 ± 1.0[cf]	16.6 ± 1.4[df]	9.7 ± 1.7[e]	6.2 ± 0.6[a]	1.1x10^{-18}
16:0	426 ± 61[a]	490 ± 21[bc]	475 ± 32[abcd]	509 ± 42[c]	444 ± 76[abd]	499 ± 50[bcd]	0.038
18:0	144 ± 21	162 ± 7	154 ± 10	166 ± 14	146 ± 25	163 ± 16	0.12
20:0	13 ± 2[a]	14 ± 1[ab]	13 ± 1[ac]	15 ± 1[b]	13 ± 2[a]	15 ± 1[bc]	0.033
22:0	15 ± 2	16 ± 1	15 ± 1	16 ± 1	14 ± 2	16 ± 2	0.19
16:1 n-7	4 ± 1[a]	7 ± 1[b]	23 ± 2[c]	24 ± 2[c]	14 ± 2[d]	5 ± 1[a]	5.3x10^{-24}
18:1 n-9 and n-7	803 ± 115	903 ± 38	857 ± 58	916 ± 76	804 ± 138	903 ± 90	0.13
18:2 n-6	2021 ± 289	2290 ± 97	2030 ± 136	2215 ± 183	1974 ± 338	2290 ± 229	0.073
18:3 n-3	248 ± 35	279 ± 12	248 ± 17	275 ± 23	244 ± 42	281 ± 28	0.078

20:5 n-3 (EPA)*	<LOD	3.3 ± 0.1[a]	35.7 ± 2.4[b]	34.3 ± 2.8[b]	22.9 ± 3.9[c]	15.7 ± 1.6[d]	$1.2x10^{-16}$
22:5 n-3 (DPA)*	<LOD	<LOD	3.5 ± 0.2[a]	3.4 ± 0.3[a]	2.9 ± 0.5[b]	1.1 ± 0.9[c]	$5.5x10^{-11}$
22:6 n-3 (DHA)*	<LOD	3.9 ± 0.2[a]	51.0 ± 3.4[b]	48.1 ± 4.0[b]	37.5 ± 6.4[c]	28.6 ± 2.9[d]	$1.7x10^{-15}$
Sodium	54 ± 8[a]	245 ± 10[b]	89 ± 6[c]	137 ± 11[d]	78 ± 13[c]	79 ± 8[c]	$4.7x10^{-25}$
Potassium	159 ± 23[a]	245 ± 10[b]	158 ± 11[a]	206 ± 17[c]	166 ± 28[a]	258 ± 26[b]	$1.1x10^{-10}$
Chloride	94 ± 13[a]	474 ± 20[b]	144 ± 10[c]	235 ± 19[d]	122 ± 21[e]	146 ± 15[c]	$4.1x10^{-27}$

Data are presented as mean ± standard deviation, n=8 in Control group, n=5 in SW-H, and n=6 in all other groups. Groups are compared using one-way ANOVA with LSD post hoc test when appropriate. Means in a row with different letters are significantly different ($p<0.05$). SW-H; Stickwater from Havstrand, PC-H; Presscake meal from Havstrand, SWPC-H; Stickwater + Presscake meal from Havstrand, PC-G; Presscake meal from Granit, FM-G; Fillet meal from Granit, LOD; level of detection. *Groups fed diets where taurine, EPA, DPA and DHA <LOD are not included in ANOVA analyses
